# Traumatic brain injury is not associated with significant myocardial dysfunction: an observational pilot study

**DOI:** 10.1186/s13049-016-0217-4

**Published:** 2016-03-16

**Authors:** Karim Serri, Malak El Rayes, Geneviève Giraldeau, David Williamson, Francis Bernard

**Affiliations:** Département de médecine, Hôpital du Sacré-Coeur de Montréal, Service de soins intensifs, Université de Montréal, 5400, boul Gouin ouest, Montréal, H4J-1C5 Canada; Centre de recherche Hôpital du Sacré-Coeur de Montréal, Université de Montréal, Montréal, Canada; Institut de Cardiologie de Montréal, Université de Montréal, Montréal, Canada

**Keywords:** Traumatic brain injury, Myocardial dysfunction, Echocardiography, Left ventricular ejection fraction

## Abstract

**Background:**

Myocardial dysfunction has been well described with catastrophic neurological events, such as subarachnoid hemorrhage and brain death. There is very limited data describing myocardial function in the context of traumatic brain injury (TBI), as no prospective study has yet examined this association. The objective of our study was to evaluate cardiac function using echocardiography in patients with clinically important TBI.

**Methods:**

We conducted a prospective observational study of consecutive TBI patients admitted to the intensive care unit. All patients older than 16 years with moderate to severe TBI according to the Glascow Coma Scale (GCS) were eligible for the study. Only patients with a prior history of heart disease or cardiomyopathy or evidence of brain death on admission were excluded. A complete transthoracic echocardiogram was performed within 4 days of admission.

**Results:**

Forty-nine patients (67 % males, median age 34 years) were included in the study. Forty-one patients had severe TBI (84 %) with a median GCS of six, 44 patients (90 %) required mechanical ventilation and 36 (74 %) intracranial pressure monitoring. Hospital mortality was 18 %. No patients had global left ventricular dysfunction as defined by a left ventricular ejection fraction (LVEF) below 50 % (95 % CI, 0–0.07). Average LVEF was 65 +/− 4 %. Four patients (8 %) had regional wall motion abnormalities with preserved LVEF.

**Discussion:**

The main finding of this study is the absence of clinically significant myocardial dysfunction in patients with moderate or severe TBI. Although myocardial dysfunction has been well described in a variety of neurological settings, it is possible that the young age of TBI patients and the absence of cardiovascular risk factors are protective against significant myocardial injury from catecholamine excess.

**Conclusions:**

In a group of patients with clinically important TBI, we did not identify any significant cardiac dysfunction.

## Background

Traumatic brain injury (TBI) is a major cause of death and disability in Western nations [1, 2]. Prognosis is dependent on damage that occurs at the time of the initial trauma, but also over the following days as secondary injury [3]. One potential contributing factor to secondary injury is autonomic dysfunction [4]. This phenomenon is thought to result from excessive adrenergic activity which most often causes abnormalities in heart rate, blood pressure and thermoregulation. In its most severe form, this excessive adrenergic activity can lead to myocardial dysfunction, which has been well characterized in the context of spontaneous subarachnoid hemorrhage (SAH). In this setting, cardiac manifestations ranging from asymptomatic electrocardiographic abnormalities to severe myocardial dysfunction [5–7] have been described and are believed to be related to catecholamine toxicity [8]. Myocardial dysfunction has also been described with other catastrophic neurological events, such as brain death in up to 40 % of patients [9–11].

There is very limited data describing myocardial function in the context of TBI, as no prospective study has yet examined this association. Based on the literature on SAH and brain death, one would expect a relatively high incidence of myocardial injury. One recent study lends support to that hypothesis with a reported incidence of 22 % of cardiac dysfunction in elderly patients with isolated TBI [12]. These findings are, however, limited by their retrospective nature and the older age of the studied population, not necessarily representative of young TBI patients.

Myocardial dysfunction in the setting of TBI could have an important impact as it may contribute to secondary injury by altering cerebral blood flow. Accordingly, the objective of our study was to evaluate cardiac function in patients with clinically important TBI. We hypothesized that cardiac dysfunction occurs frequently after TBI and that it is associated with the severity of the TBI.

## Methods

### Setting

We conducted a prospective observational study of consecutive TBI patients admitted to the intensive care unit (ICU) at Hôpital du Sacré-Coeur de Montréal, a 500-bed general hospital in Montreal, Canada. The institution is an academic level 1 trauma center affiliated with the University of Montreal, with more than 1600 trauma admissions per year and between 150 and 180 moderate or severe TBIs per year. The unit is an intensivist-run unit with 38 ICU beds, among which 10 are dedicated for neurological ICU patients. The study was approved by the local institutional review board (CER, Hôpital Sacré-Coeur de Montréal). Informed consent was obtained from the patients’ next-of-kin.

### Study population

All patients older than 16 years with moderate to severe TBI according to the Glascow Coma Scale (GCS) were eligible for the study. TBI was categorized as moderate (GCS 9–12) and severe (GCS 3–8) according to Brain Trauma Foundation guidelines. Only patients with a GCS of less than 13 were included in the study. Patients with a prior history of heart disease or cardiomyopathy and patients with evidence of brain death on admission were excluded. Patients whose ultrasound images were deemed uninterpretable were also excluded from the study.

### Patient care

Patients were treated according to local institutional practices consistent with the Brain Trauma Foundation guidelines [13–15]. Intracranial pressure (ICP) monitoring was performed in patients in whom it was clinically indicated as per the treating neurosurgeon and intensivist. In patients with ICP monitoring, cerebral perfusion pressure was kept above 60 mm Hg while maintaining ICP below 20 mm Hg. Mean arterial pressure was measured with an arterial line calibrated at the level of the tragus. PaCO2 was kept between 4.7–5.3 kPa (35–40 mm Hg) in mechanically ventilated patients, and O2 saturation > 94 %. Efforts were made to keep patients euvolemic, to maintain natremia and glycemia normal and to prevent hyperthermia. Analgesia and sedation were used at the minimal required doses to allow optimal ICP control. Cerebral computed tomography (CT) scans were performed on admission and as needed thereafter.

### Outcome measures

Demographic, clinical, laboratory and electrocardiographic data were collected at the time of admission. Standard twelve lead electrocardiograms (ECG) were recorded daily for the first 4 days following admission. Troponin I levels were drawn daily for the first 4 days after admission with a level greater than 0.1 mcg/l considered abnormal. CK, CK-MB levels were measured as well. TBI severity was graded clinically according to the GCS after resuscitation and radiographically according to the Marshall [16] and Rotterdam scores [17]. Measurement of ICP was documented when available. An elevated ICP was defined as a non-stimulated spontaneous increase greater than 20 mm Hg for more than 5 min.

A complete transthoracic echocardiogram was performed within the first days of admission, with assessment of left ventricular (LV) function and dimensions according to current guidelines [18]. The images were acquired in 5 standard views (parasternal long and short axis, apical 2-, 3- and 4-chamber) and stored digitally in cineloop format using a commercially available ultrasound system. LV ejection fraction was assessed visually and measured by the Simpson method when possible. LV diastolic function was assessed as recommended by the American Society of Echocardiography by calculating E/A and E/e’ ratios [18]. Briefly, using pulsed-wave Doppler, the early mitral filling wave (E) and the atrial contraction wave (A) were measured and the E/A ratio was calculated. Using tissue Doppler, the early mitral filling wave (e’) was measured at the lateral mitral annulus and the E/e’ ratio was calculated. Right ventricular function was evaluated by measuring tricuspid annular velocity (S’) by tissue Doppler [19]. LV dimensions, left atrial dimensions and the presence of valvular disease were assessed according to recommendations. Two independent blinded experienced observers unaware of patient clinical condition reviewed all echocardiograms.

The main study outcome was the presence of LV dysfunction as defined by a LVEF < 50 %. As secondary outcomes, we measured troponin levels and studied ECG patterns to determine if there was an association with LV dysfunction.

### Statistical analysis

Based on data from SAH patients and brain death patients, we estimated the prevalence of myocardial dysfunction to be approximately 20 %. We planned to enroll 50 patients in this pilot study, which would have allowed to detect myocardial dysfunction with a precision of 11 % with a confidence interval of 95 %. Continuous data is presented as mean ± SD or median (IQR) according to distribution and categorical data is presented as proportions.

## Results

### Baseline characteristics

Between October 2009 and January 2012, a total of 64 patients were screened and 49 patients were included in the study. Exclusions were mainly due to poor ultrasound image quality. Baseline characteristics are presented in Table 1. The majority of patients were men (67 %) with a median age of 34 years (IQR 27.5, range 16–81). Four patients were older than 65 years, and 6 were older than 50. The mechanism of TBI was most frequently a motor vehicle accident, accounting for 58 % of the cases, with the remainder being due to falls, fights, and falling objects. Forty-one patients had severe TBI (84 %). The mean GCS was 6 +/− 3 (range 3–13). Thirty-six patients (74 %) required ICP monitoring, and 12 had an elevated ICP (25 %). Twenty-four patients (49 %) had SAH and 20 (41 %) had a subdural hematoma. Thirteen patients (27 %) required a craniectomy. Associated traumatic injuries were the following: 12 patients with orthopedic fractures, 12 with maxillofacial fractures, 7 with spinal fractures, and 13 with other injuries (the vast majority representing pulmonary contusions). Thirty-seven patients (76 %) received vasopressors (none received inotropes) and 44 (90 %) were mechanically ventilated. There were 9 deaths during the index hospital admission (18 %).

**Table 1:** Baseline characteristics

Median age (range)	34 (16–81)
Male (%)	67
Mean GCS	6 (+/− 3)
Severe TBI (%)	84
ISS	32 +/− 11
Subarachnoid hemorrhage (%)	49
Subdural hematoma (%)	41
Mean Marshall score	2.8 +/− 1.5
Mean Rotterdam score	3.5 +/− 1.2
ICP monitoring (%)	74
Craniectomy	27
Vasopressors (%)	76
Mechanical ventilation (%)	90
Mortality (%)	18

*GCS* Glasgow Coma Scale, *TBI* Traumatic brain injury, *ISS* Injury Severity Score, *ICP* Intracranial Pressure

### Echocardiographic results

Echocardiography was performed at a median of 49 h (8–189) post trauma, and 18 patients had their echochardiograms within 48 h. Table 2 shows the results of the echocardiographic findings. LV systolic and diastolic dimensions were normal. The average LVEF was 65 +/− 4 % (*n* = 49). No patients had global LV dysfunction as defined by a LVEF below 50 % (95 % CI, 0–0.07). Four patients (8 %) had regional wall motion abnormalities with preserved LVEF. Diastolic function was normal in 39 patients, could not be determined in eight patients, and two patients had mild diastolic dysfunction. The estimated left ventricular filling pressures were normal with an average lateral E/e’ of 5.7 +/−1.5 (*n* = 39) (normal E/e’ < 8). No patient had right ventricular dysfunction. Five patients were found to have mild valvular regurgitation, no patient had significant valvular abnormalities.

**Table 2:** Echocardiographic findings

	Study patients	Reference range
LVEF (%)	65 (+/− 4)	≥55
LVEDD (mm)	46 (+/− 6)	F 38–52, M 42–58
LVESD (mm)	30 (+/− 6)	F 22–35, M 25–40
E/A	1.5 (+/− 0.5)	0.8–1.5
Lateral E/e’	5.7 (+/− 1.5)	≤8
Right ventricular S^’^ (cm/s)	15 (+/− 3)	≥10

*LVEF* Left ventricular ejection fraction, *LVEDD* Left ventricular end-diastolic dimension, *LVESD* Left ventricular end-systolic dimension, *E* Early filling wave, *A* Atrial contraction wave, *e’* Tissue doppler early filling wave, *S’* Tissue Doppler tricuspid annular velocity

### ECG and troponin

Troponin I levels and CK-MB levels were available in 43 and 40 patients respectively. Fifteen patients (31 %) had an elevated troponin level. The median maximum troponin I concentration was 0.05 mcg/l (IQR 0.22, range 0.01–22). Maximal troponin elevation occurred at an average of 1.9 days. The median maximal CK-MB was 15 U/l (IQR 21.7, range 2.8–212.4). Of the four patients with regional wall motion abnormalities, none of them had elevated troponin levels, and none of these patients died. As for cardiac risk factors, three patients had hypertension, one was a known diabetic, and eight were smokers.

ECG abnormalities were present in 28 patients (57 %), for the most part non specific ST segment or T waves changes. Five patients had ECG changes suggesting acute ischemia, none of which had enzymatic criteria for myocardial infarction or required coronary angiography. QT interval was prolonged in 22.4 % of patients, with severe prolongation (QTc > 500 ms) in 8 %. Average QTc was 456 +/− 43 ms.

## Discussion

The main finding of this study is the absence of clinically significant myocardial dysfunction in patients with moderate or severe TBI. Although troponin elevation and ECG abnormalities are frequent, they do not appear to be associated with myocardial dysfunction. This is to our knowledge the first prospective study of myocardial dysfunction and TBI.

Myocardial dysfunction has been well described in a variety of neurological settings. Aneurysmal SAH has been the most studied example with impaired systolic function reported in 8–53 % of patients. In an echocardiographic study of 300 patients with SAH, Kothavale et al. described wall motion abnormalities in 18 % of patients and in 35 % of patients with high-grade SAH [5]. A reduced LVEF (<50 %) was found in 8 % of patients. In a study of 294 patients with SAH, Urbaniak et al found impaired systolic function (LVEF < 40 %) in 53 % of patients [6]. The mechanism responsible for myocardial injury in this setting is thought to be catecholamine toxicity [8, 20, 21]. The sympathetic discharge associated with SAH causes a specific form of myocyte injury called contraction band necrosis [22]. These findings have been noted both in experimental and human studies [23–25]. Similar pathophysiologic mechanisms may be involved in stress cardiomyopathy and brain death-associated myocardial dysfunction [26, 27].

Though well described in these settings, there is very limited data in patients with TBI, as only two retrospective studies have evaluated the association between TBI and cardiac function. In a preliminary retrospective study of 51 patients with severe brain injury in whom transesophageal echocardiography was performed, LV dysfunction was observed in 14 % of patients, and severe LV dysfunction in 8 % [10]. Another 16 % of patients were found to have regional wall motion abnormalities with preserved global LV function. Of note in this study, 45 % of patients had non traumatic brain injury and all patients eventually evolved towards brain death. Although brain death diagnosis was not yet made at the time of the echocardiogram, it is possible that some patients may already have been brain dead. Furthermore, because of the retrospective nature of the study, it was not possible to determine if patients had pre-existing heart disease. A recent retrospective study of 139 patients with TBI reported abnormal echocardiograms in 22 % of patients and reduced LVEF in 12 % of cases [12]. There are however significant differences with the present study: patients were older (58 years on average) and the predominant traumatic lesions were subdural hematomas (63 %). Older age and greater prevalence of cardiovascular risk factors could explain some of the echocardiographic abnormalities. Furthermore, the fact that echocardiograms were not part of a protocol but ordered on clinical grounds, and the retrospective nature of the study could have introduced significant selection bias. For these reasons, cardiac dysfunction may have been overestimated.

We did not observe any significant myocardial dysfunction in our patient population. All parameters of cardiac function, including systolic and diastolic function, left and right ventricular function, were normal. Diastolic function is often an early marker of myocardial dysfunction appearing before any systolic abnormalities. Taken together, these findings are consistent overall with the absence of any clinically significant cardiac dysfunction following TBI. Although no prospective human data is available, a recent animal study did not identify any functional or histopathological abnormalities in a rat model of TBI [28]. Troponin elevation however was frequent in our patients, but was not associated with TBI severity or myocardial dysfunction. In a retrospective review of 420 patients with TBI, Salim et al. found increased troponin I levels in 29.8 % of patients using a cut-off of 0.3 ng/ml, very similar to our findings. The authors describe an association between troponin levels and severity of brain injury as well as outcome. Interestingly, beta-blocker use was found to be protective [29]. We observed an elevated proportion of ECG abnormalities in our patients, for the most part non specific changes and QT prolongation. These findings are similar to what has been described in SAH [30].

The reason why other forms of brain injury are associated with cardiac dysfunction and not TBI is unclear. As mentioned above, brain-heart interactions have been well documented in SAH. Severity of injury does not appear to play a role, as our study mostly involved patients with severe TBI. Considering the pathophysiology of myocardial dysfunction in SAH, one could postulate that TBI be associated with lower levels of catecholamines, which again appears unlikely considering the severity of disease. However, it is possible that the younger age of TBI patients (and the associated absence of cardiovascular risk factors) compared to typical SAH patients is protective against significant myocardial injury from catecholamine excess.

### Limitations

The main limitations of our study is the small sample size, which may have limited the power to detect an association between TBI and significant cardiac dysfunction. However, none of the echocardiographic parameters were in favour of myocardial dysfunction. Studies were performed 49 h post trauma, which may have been late to identify very early myocardial dysfunction. Based on data on SAH patients, one would expect cardiac dysfunction to persist at least several days [31]. Hence, if myocardial dysfunction occurs very early in TBI, it does not appear to be clinically significant.

## Conclusion

Although troponin elevation and ECG abnormalities are frequent in patients with moderate to severe TBI, we did not identify any significant cardiac dysfunction. It is possible that the young age of patients with TBI and the absence of cardiovascular risk factors are protective against significant myocardial injury from catecholamine excess. Further studies to confirm these findings and to understand the underlying pathophysiology are needed.

## Abbreviations

ECG: electrocardiograms
GCS: Glascow Coma Scale
ICP: Intracranial pressure
ICU: intensive care unit
LV: left ventricular
LVEF: left ventricular ejection fraction
SAH: subarachnoid hemorrhage
TBI: traumatic brain injury
